# Integrating Protein Interaction Surface Prediction with a Fragment-Based Drug Design: Automatic Design of New Leads with Fragments on Energy Surfaces

**DOI:** 10.1021/acs.jcim.2c01408

**Published:** 2022-12-27

**Authors:** Luca Torielli, Stefano A. Serapian, Lara Mussolin, Elisabetta Moroni, Giorgio Colombo

**Affiliations:** † Department of Chemistry, University of Pavia, Via Taramelli 12, Pavia27100, Italy; ‡ Department of Woman’s and Child’s Health, Pediatric Hematology, Oncology and Stem Cell Transplant Center, University of Padua, Via Giustiniani, 3, Padua35128, Italy; § Istituto di Ricerca Pediatrica Città della Speranza, Corso Stati Uniti, 4 F, Padova35127, Italy; ∥ SCITEC-CNR, via Mario Bianco 9, Milano20131, Italy

## Abstract

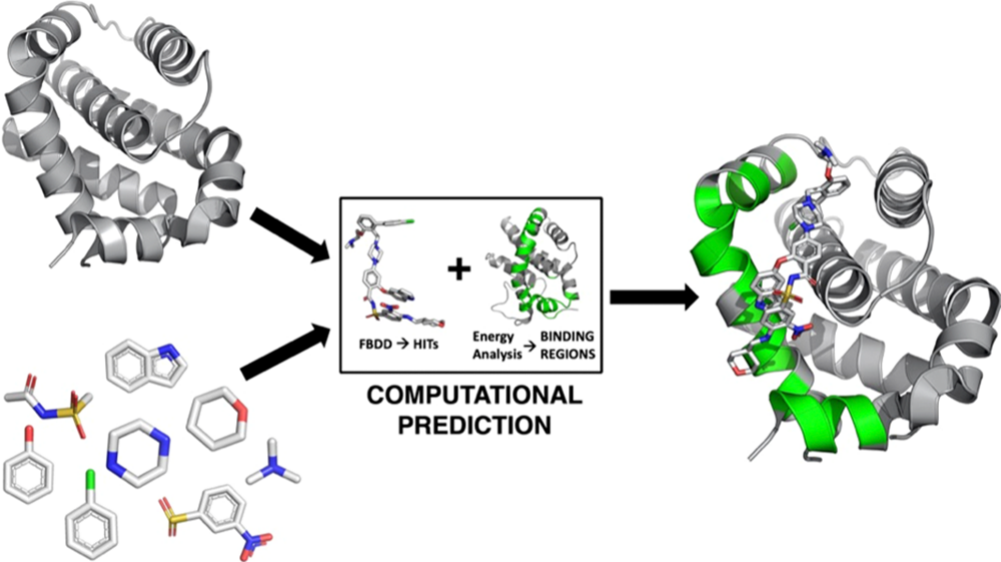

Protein–protein interactions (PPIs) have emerged in the past years as significant pharmacological targets in the development of new therapeutics due to their key roles in determining pathological pathways. Herein, we present fragments on energy surfaces, a simple and general design strategy that integrates the analysis of the dynamic and energetic signatures of proteins to unveil the substructures involved in PPIs, with docking, selection, and combination of drug-like fragments to generate new PPI inhibitor candidates. Specifically, structural representatives of the target protein are used as inputs for the blind physics-based prediction of potential protein interaction surfaces using the matrix of low coupling energy decomposition method. The predicted interaction surfaces are subdivided into overlapping windows that are used as templates to direct the docking and combination of fragments representative of moieties typically found in active drugs. This protocol is then applied and validated using structurally diverse, important PPI targets as test systems. We demonstrate that our approach facilitates the exploration of the molecular diversity space of potential ligands, with no requirement of prior information on the location and properties of interaction surfaces or on the structures of potential lead compounds. Importantly, the hit molecules that emerge from our ab initio design share high chemical similarity with experimentally tested active PPI inhibitors. We propose that the protocol we describe here represents a valuable means of generating initial leads against difficult targets for further development and refinement.

## Introduction

Protein–protein interactions (PPIs) oversee a wide range of fundamental functions in cells, from protein folding to protein degradation, from signal transduction to transport processes, and from enzyme regulation to the regulation of DNA biochemistry.^1−9^ The perturbation of physiological PPIs, determined by ligand binding or protein modifications caused by possible external stresses, reverberates into the malfunction of functional assemblies, which ultimately leads to disease states.^9−12^

Therefore, it comes as no surprise that PPIs have emerged as a new and attractive class of molecular targets for drug discovery and development. Indeed, blocking PPI malfunctioning in transformed cells (while leaving normal cells unperturbed) could represent an optimal strategy for the treatment of many pathological conditions.^13,14^

However, PPIs are challenging targets for pharmacological interventions. They are in fact characterized by the heterogeneity of their sizes and shapes: typically, the areas involved in interactions exceed 4000 Å^2^, making them complicated to engage them with small molecules.^13,14^ Moreover, a feature that distinguishes these targets from classical active or binding site proteins is the absence of well-defined pockets and cavities, which would facilitate the design of ad hoc molecules, using established methods [e.g., docking, high-throughput screening (HTS), pharmacophore analysis, etc.]. Finally, because these large surfaces are largely apolar, the interactions that a ligand could form could expectedly be hydrophobic and thus potentially weak or even a-specific.^15−18^

For all these reasons, there are currently a limited number of active ligands for PPIs, and these targets have often been defined “undruggable”.

To overcome the hurdles described above, the preferred approach to interact with large surfaces has involved the use of oligopeptides and peptidomimetics. Recent years have witnessed the impact of PROTACs, a new class of bidentate drugs able to recruit (often undruggable) proteins to the unfolded protein response machinery of the cell.^8,19−25^

Fragment-based drug design (FBDD) also recently emerged as an attractive strategy to design ligands able to engage the large protein surfaces involved in PPIs. In this approach, the target is probed with low-molecular-weight ligands (∼150 Da).^26−28^ Their 3D binding mode can be determined via X-ray crystallography and NMR spectroscopy.

Information on the fragments identified to bind productively is then used to guide their evolution into optimized molecules with drug-like properties.^29,30^ This can be achieved either by growing a larger molecule out of a binding fragment or by connecting different fragments that bind to distinct areas of the interaction surface. A successful example of this approach is represented by the design of Venetoclax, drug approved by the FDA in 2016.^31^

In the last few years, computational approaches have made a significant contribution to PPI targeting. In general terms, they encompass methods for the prediction of the PPI to be targeted and for the evaluation of the potential binding of candidate HITS. The former includes coevolution analyses, homology modeling, and multiple sequence alignment: many of these are currently being coupled to machine learning approaches. The latter entails mainly docking methods to search for potential ligands from databases and define their putative binding modes using physics-based energy functions or geometric models. Many of these methods are based on the use of pre-existing information on the protein or sequence to be targeted. Docking methods, on the other hand, may be limited in their efficiency by the extension and complexity of the surfaces that need to be scanned. In this context, it is worth underlining that computational methods have been developed that couple the selection of possible fragments to the exploration of the potential binding surface using molecular dynamics (MD) simulations and enhanced sampling techniques.^32−36^

**Figure 1:**
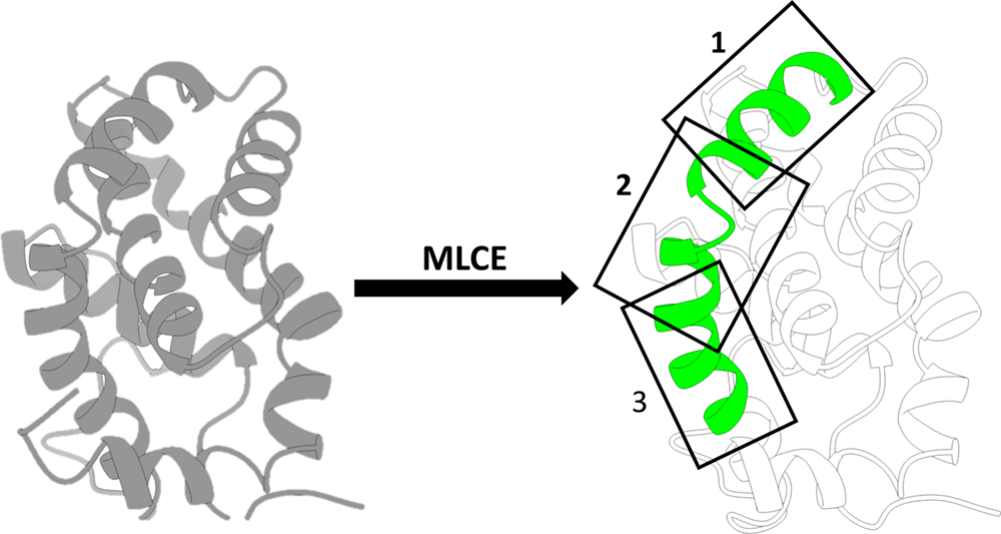
Schematic representation of the FOES approach. MLCE identifies a portion of protein’s surface. The latter is then subdivided into sectors (1, 2, and 3) which are then scanned via drug-like fragments.

Here, we present fragments on energy surfaces (FOES), a simple and straightforward (ab initio) approach that facilitates the identification of new hit molecules to engage specific PPIs, independent of pre-existing information on the target and possible ligands. To this end, we use as input only the 3D structure in isolation of one of the PPI-forming partners to target and a small library of fragments. As a test set, we choose several structurally different disease-related proteins which are known to carry out their activity via the assembly of specific PPIs. First, knowledge on the 3D structure of the target protein is used to predict the location of potential interaction regions using our recently introduced energy-based method for PPI interface identification.^37−40^ Next, the putative binding surface is subdivided into overlapping windows (Figure 1): each window is used as a target for fragment docking. Finally, the fragments with the best scores are automatically connected via simple chemical groups (such as methylenes in the simplest scenario explored here) to form new HIT compounds. FOES is validated by comparing, via a chemical and structural similarity-based score, the structures of the designed HIT compounds with those of molecules that have been experimentally proven to successfully bind the target compounds, for which a crystal structure in complex with the protein of interest exists.

Upon analyzing the generated compounds and benchmarking against experimental complexes containing PPI-targeting ligands, we show that a high degree of similarity with known actives naturally emerges for designed HITS. Importantly, this is achieved with no prior information on the chemistry of the ligands or on the location of the surfaces. The HITs identified through FOES are clearly non-optimized and can represent valuable starting points for ad hoc medicinal chemistry campaigns.

FOES is fully general and immediately applicable to new targets in the context of PPI studies. Furthermore, we expect it to be useful to unveil possible hits for orphan proteins, often very important for disease, but generally difficult to treat.

## Methods

### MLCE

The prediction of binding regions on selected proteins was carried out using the matrix of low coupling energy (MLCE) method. MLCE starts from the analysis of the pair-interaction energies of all the amino acids in a protein.^41−45^ The method has been described at large previously and tested experimentally in various settings. Here, we provide a brief overview. First, MLCE computes the non-bonded part of the potential *E* (van der Waals, electrostatic interactions, and solvent effects) via a MM/GBSA calculation, obtaining, for a protein of *N* residues, a *N* × *N* symmetric interaction matrix *M_ij_*. This matrix can be expressed in terms of its eigenvalues and eigenvectors as
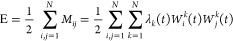
1where λ_K_ is the *k*th eigenvalue and *w_i_^k^* is the *i*th component of the corresponding eigenvector. The eigenvector associated with the most negative eigenvalue contains information on the most and least stabilizing interactions in the system.

It was previously shown that the first eigenvector contains most of the relevant energetic information on interactions of the system, and we can define an approximated interaction matrix *M̃*_*ij*_ as
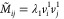
2

Under the assumption that residues involved in structural stability, corresponding to the components with highest values in *v*^1^, are not prone to adaptation, change of conformation, and dynamic behavior, we consider the binding interaction to a potential partner as a local phenomenon involving regions not directly dedicated to structural stabilization. From this point of view, we can filter the approximated interaction matrix *M̃*_*ij*_ to contain only the pairs of residues that are in geometric proximity in the analyzed structure, obtaining the MLCE. Namely,
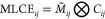
3where *C_ij_* is the residue contact matrix, which is equal to 1 if the residues *i* and *j* are closer to 6.5 Å (in this implementation, we consider C_β_ for proteins and C1 for glycans) and 0 otherwise, and ⊗ is the Hadamard product (i.e., element-by-element product).^39^

Starting from the MLCE_*ij*_ matrix, we select the residues that have an interaction energy with respect to the other residues that are smaller than a threshold. In practice, with a threshold of 15% (the default value), we select the residues involved in the 15% of non-zero interactions that are less energetic. This threshold value proved to be a key parameter for specificity/sensitivity of the approach. The residues obtained are then merged in patches that are sets of residues that are close to each other and constitute the predicted protein–protein binding regions.^39^

Specifically, MLCE calculations are applied to structural representatives obtained from the MD simulations of Bcl, VHL, and HIV integrase (vide infra). The setup of MD simulations and the selection of structural representatives via clustering are described in the next paragraphs.

#### Protein Preparation

The proteins were downloaded from the Protein Data Bank with the following PDB codes: Bcl, 1GJH.pdb; VHL, 4AJY.pdb; HIV, 3L3U.pdb. Possible detergent molecules, ligands, etc., were removed before entering the preparation protocol. The structures of the proteins were first refined using the Protein Preparation Wizard tool of Maestro suite of programs (www.schrodinger.com).^46^ This tool assigns correct bond orders, adds missing hydrogens, and creates disulfide bonds where needed/possible. During the process, the pH used was set between 6 and 8. The protonation state of acidic/basic residues was assigned using the PROPKA tool at pH 7. The resulting structures were subjected to a gentle backbone-restrained minimization.

#### MD Simulation

The resulting structure for each protein was used as the input for a 1 μs MD simulation. All MD simulations were carried out with the DESMOND module of the Maestro suite. First, a cubic box was used as a solvation box: in all cases, the boxes were built large enough to allow a distance of 1 nm between the edge of the box and the surface atoms of the minimized structure of the protein. The box was filled with the solvent TIP3P water molecules, and the complex was brought to neutrality by the addition of sodium or chlorine ions depending on the total charge of the protein.

The simulation was next prepared as follows:

1. A Brownian dynamic was run, in an *NVT* environment, at a temperature of 10 K with a timestep of 1 femtosecond and restraints on solute heavy atoms. The total duration of this step is 100 picoseconds.
2. A subsequent step in an *NVT* environment was run at a temperature of 10 K with a timestep of 1 femtoseconds and restraints on solute backbone heavy atoms. This time the duration was 12 picoseconds.
3. This step was run in an *NPT* environment, starting at a temperature of 10 K and restraints on solute heavy atoms, for 12 picoseconds. In this step, the temperature is gently raised to 100 K.
4. The same as step 3, but the temperature is progressively raised to 300 K.
5. In this step, the *NPT* environment with no restraints is used for 24 picoseconds to allow the system to smoothly adapt to the 300 K temperature condition.
6. Starting from the final structure obtained at the previous step, the production MD simulation is run for 1 microsecond for each system.

The velocities and coordinates of each simulation were generated randomly, the simulation was run in a periodic system, the barostat used was the Langevin barostat (1 Bar), and the thermostat was the Nose–Hoover chain (300 K). All simulations were carried out with DESMOND (www.schrodinger.com),^47^ with the S-OPLS force field.

#### Clustering

A clustering analysis using the hierarchical clustering algorithm (implemented in MAESTRO) was carried out on each of the resulting trajectories for each of the systems. We selected the representative structures from the six most populated clusters.

For each protein, the binding region was considered as the consensus result by applying MLCE to the minimized X-ray structure and the six structures identified by the clustering analysis.

#### Docking

To perform the docking study on the previously chosen structure, binding windows were constructed on the portions of the protein found by the MLCE analysis.^39^ This division into windows is intended to divide very large portions of surfaces into smaller portions in order to sample the entire region with fragments as exhaustively as possible (Table 1).

**Table 1:** Here, the Various Combinations of Residues Selected To Build the Grid for Docking and To Assess the Entire Binding Windows on the Surfaces of Proteins Are Reported^a^

Bcl	
1	60–75
2	57–65, 94–101
3	39–51, 146–164

VHL	
1	13–16, 49–52, 88–89
2	15–20, 46–50, 88–92
3	19–20, 46, 92–94

HIV	
1	33–40, 62–68
2	31–37, 116–120, 183–196
3	163–167, 182–185, 259–268
4	38–51, 68–76, 259–271

VHL	
1	13–16, 49–52, 88–89
2	15–20, 46–50, 88–92
3	19–20, 46, 92–94

HIV	
1	33–40, 62–68
2	31–37, 116–120, 183–196
3	163–167, 182–185, 259–268
4	38–51, 68–76, 259–271

a See the main text for the definition and graphical representation of the windows.

Docking was carried out with the Glide module of the Maestro suite of programs. We used the same setup for each case:^48^ In XP precision docking, 10,000 poses were generated for each fragment in the first docking phase, and of these, the best 1000 were kept (based on the energy score). Eventually, only the best pose is saved as output.

At the end, another docking study was done on the raw HITs molecules to verify that they were akin to the chosen interaction site using the same settings.

#### Fragment Combination

After selecting only those fragments that interacted with the protein in the identified region and gave interactions characteristic of small-molecule–protein bonds, the Combine Fragments tool was used to achieve fully computational and automated linkage between fragments. To do this, different strategies were used depending on the case study:

1. In the case of VHL and HIV, the selected fragments were all treated the same way: automatic “direct joining” (random) was done between the fragments considered.
2. In the case of Bcl, considering a much larger surface to cover, a core was created with the two fragments that bound to the mid-low portion of the helix of interest. First, these two fragments were prepared with LigPrep individually to see which were the most likely stereoisomers. After that, the four result structures of the two starting fragments were combined with the Combine Fragments tool. The result of this core (having decine of different starting cores available) was used with the starting structure for the construction of the final molecules: using the “linking” option, and entering the cores, two “number of trials” were made by adding from one to three fragments.

For both strategies used, settings were used to allow all the chosen fragments to be considered. To do this, allowed distances and angles were adjusted specifically according to the protein being studied.

#### Fingerprint Similarity (Tanimoto)

As a final analysis, a similarity study was done using the Tanimoto metric^49^ as implemented in Maestro. The following settings were used for this analysis:

- 64-bit precision
- Fingerprint type atom pairs
- Atom typing scheme 4
- Similarity metric Tanimoto

### Fragments

The fragment library was taken from Schrödinger’s site and prepared with the latest force field through the LigPrep tool using the default settings. A preparation step was first run, and the OPLS4^50^ force field was used considering a pH between 6 and 8 using Epik.

## Results

### General Scheme of FOES

In this study, we considered several structurally unrelated proteins that are known to carry out their functional activity via the formation of complexes with other protein partners and to be important pharmacological targets. We studied Bcl,^51^ VHL,^27^ and HIV integrase.^52^ These proteins are structurally different and are involved in distinct disease pathways. For all targets, we started from the experimental (X-ray or NMR) structure of the protein in isolation and removed possible bound ligands, detergent molecules, trapped solvents, etc. Each protein was then simulated for 1 μs using all-atom MD simulations. This step is intended to relax the structure and remove biases/correlations with the initial structure. From the resulting trajectory, the six most representative structures are extracted as the centers of the six most populated conformational clusters. These were then used together with the initial minimized structure (thus giving seven target structures in total) as the basis for the blind prediction of potential protein interaction surfaces.

To this end, we used the MLCE approach.^37−40^ In this framework, we analyze the energetics of residue-pair interactions as this can unveil key information on the structural organization and localization of interacting areas of the molecule. The working hypothesis is that specific networks of residues may be dedicated to fold stabilization, while others may deal with establishing interactions with partners. Evolutionary pressure has in fact selected protein–protein binding sites favoring those chemical and conformational properties that guarantee the correct function. Internal energetics accounts for the interactions that each residue establishes with all other residues of the protein it belongs to: in this context, strong pair interactions identify internal residues related to the stabilization of the folding core, while weaker pair interactions, combined with the localization of residues in continuous patches on the protein surface, highlight substructures that are not internally optimized and are thus prone (or in other words preorganized) to interact with a potential partner.

In this spirit, MLCE analyses of the interaction energies of all the amino acids in a protein compute the non-bonded part of the potential (van der Waals, electrostatic interactions, solvent effects) via a MM/GBSA calculation, obtaining, for a protein composed of *N* residues, a *N* × *N* symmetric interaction matrix *M_ij_*. The eigenvalue decomposition of the matrix highlights the regions of strongest and weakest couplings: the fragments that are on the surface, contiguous in space and weakly coupled to the protein core, define the potential interaction regions. Putative interaction patches can be considered frustrated (non-optimized) in terms of intramolecular interactions and open to stabilization by partners. MLCE has been extensively validated, also in experimental contexts.^39,41−43,53,54^

The seven representative structures for each protein are thus analyzed with MLCE, and the consensus results highlighting non-optimized substructures that are consistently found in distinct clusters were selected as the regions that could aptly be targeted by small drug-like fragments. As the predicted surfaces are expectedly large, they are first subdivided into overlapping regions, or windows (Figure 1), to reduce the complexity of configurational and conformational searches in fragment docking. This allowed us to accurately and efficiently sample the entire putative interaction portion found by MLCE analysis.

Through the analysis of the location, energetics, and interaction patterns of the fragments with their respective target areas, the most promising ones are selected.

Once the most relevant fragments are selected, they are joined in the simplest way possible using the Combine Fragments tool (see Methods) of the Schrodinger MAESTRO suite (www.schrodinger.com): directly, with one CH_2_ or with two CH_2_ bridging moieties. The aim here was to simplify the construction process as much as possible.

**Figure 2:**
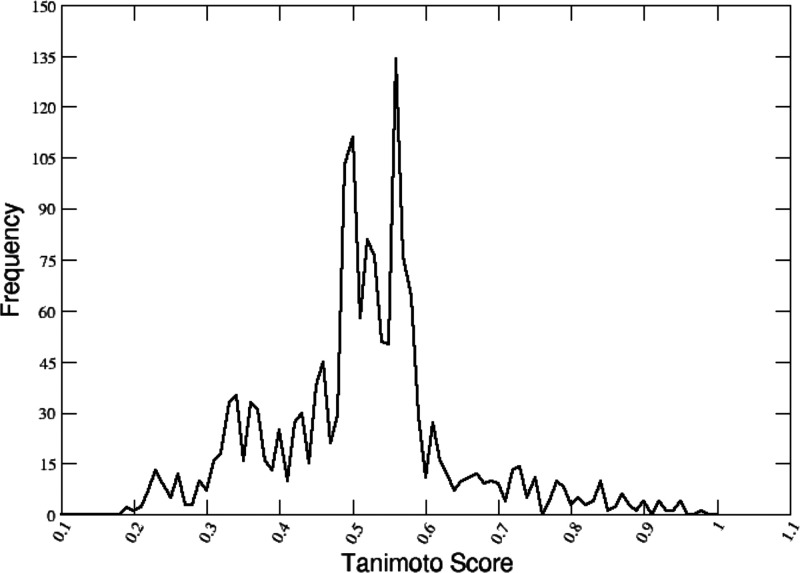
Overall distribution of similarity scores. The graph reports the overall distribution of the Tanimoto similarity scores calculated for all the molecules created with the FOES algorithm. Similarity scores are calculated using experimentally characterized ligands targeting the same proteins.

After obtaining these initial combined HITs, the similarity between the generated molecules and the structures of known (active) ligands for the respective proteins (of which there was a ligand-protein co-crystal) was calculated. To do this, the Tanimoto similarity metric^49^ was used (Figure 2) based on the Fingerprint similarity tool in the Schrodinger MAESTRO suite (see Methods).

In this context, one should keep in mind that a key problem in comparing the generated HIT and the experimentally tested ligand resides in the different size of the molecules being compared. Specifically, the reference molecule may have a different number of features from either the fragments or the fully reconstructed HITs. As a consequence, even if the main features were correctly captured by the fragment-based approaches, since they would likely represent a subset of the features present in the benchmark molecule, one may expect to obtain low similarity coefficients. Moreover, in our approach, we use no information on the cryptographically determined binding region: to identify the location of the interaction surface, we indeed use structures that are generated by MD simulations. In this framework, the actual shapes and conformations displayed by the protein for interactions may differ from the ones observed in the structures we use as benchmarks, favoring the generation of HITs with conformations that are different from the ones observed for X-ray characterized experimental ligands in complex with the protein used as benchmarks. To overcome these potential limitations, we used a variant of the Tanimoto similarity score implemented in the MAESTRO suite, which compares atom pairs taking their relative distances in space into account, coupled to “atom typing scheme 4” which takes into account functional groups and their bonding hybridization. The scores obtained are reported in the form of graphs and tables throughout the paper (vide infra).

Finally, we ran a docking study using designed HITs to characterize the potential affinity of the molecules for their respective target protein. Strikingly, the Hits with the highest calculated affinities were shown to belong to the ensemble of structures with the higher similarity coefficients to known active ligands.

### Bcl

Bcl proteins are a family of proteins involved in the regulation of programmed cell death (apoptosis). Alteration of their expression in cancer makes them oncogenic promoters as their task of switching on the programmed death of cancer cells is suppressed.^55^ This family of proteins forms an extensive interactions network within the cytosol and membranes.^56^

**Figure 3:**
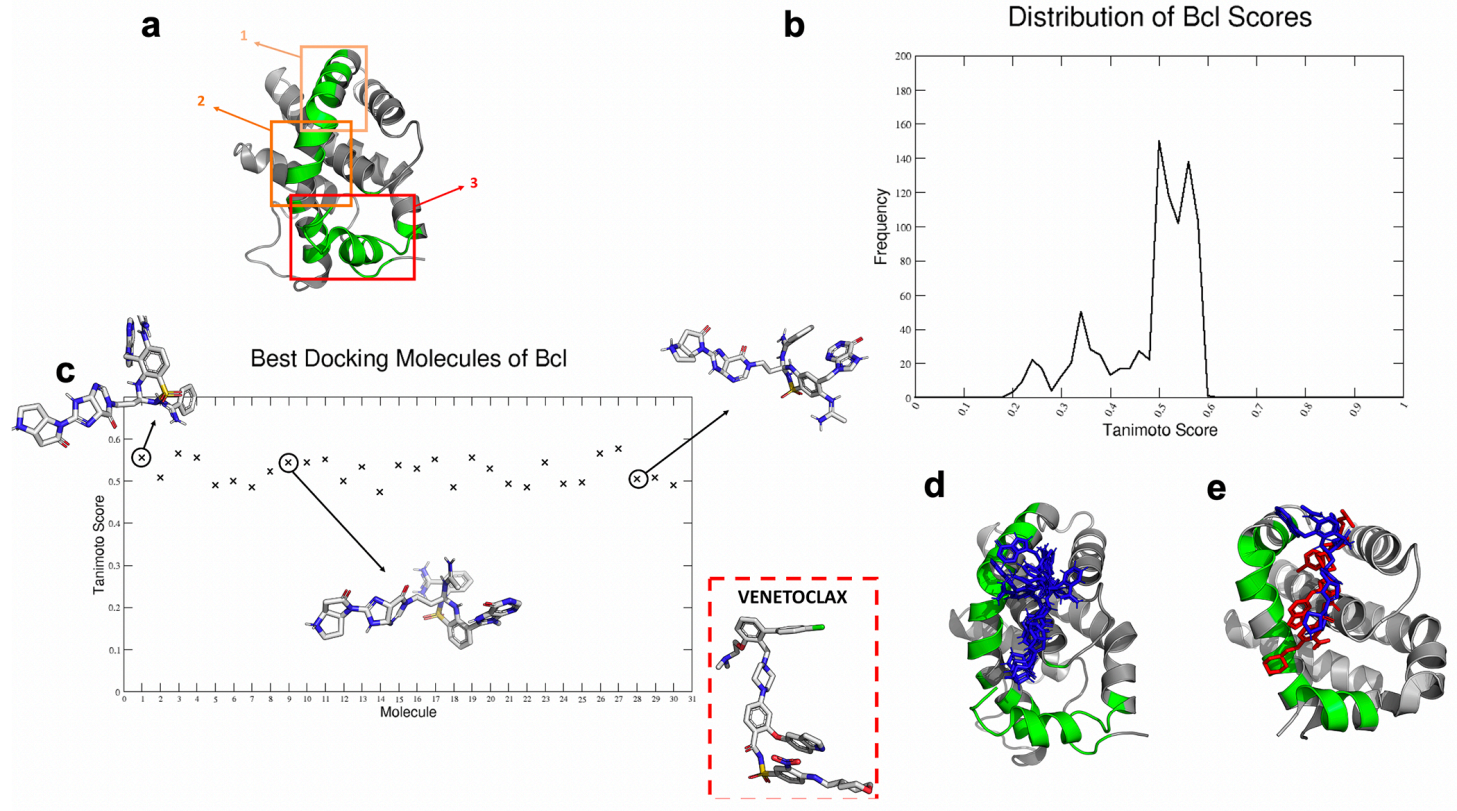
Development of HITs for Bcl-2. (a) Docking sectors built for Bcl based on the result of MLCE analysis: the predicted binding region was divided into three parts. Sector 3 includes the C-terminal helix. (b) Distribution of the Tanimoto similarity scores, described in the main text, for all the Bcl-binding HITs generated against Bcl vs Venetoclax, the active drug against Bcl. (c) Tanimoto scores of the 30 best molecules (reported on the *X*-axis) derived from final docking analysis. The three molecules shown are some example of the ligands found with FOES. In the box, the original ligand (Venetoclax). (d) The best docking poses on Bcl of the five best ligands built with FOES. Focusing on the green areas, it is clear that the FOES-generated ligands engage the same portion of the PPI-helix as that involved in the binding with Venetoclax (see 3e). (e) Red color indicates Venetoclax, and blue color indicates one of the representatives of best docking generating HITs. The figure highlights the optimal overlap between the two molecules.

We first applied MLCE to identify possible interaction surfaces on Bcl (Figure 3a). The consensus analysis on different clusters returned the α-helix spanning residues 46–76 and 151–164 (PDB code: 1GJH) as the most probable binding region. This was then taken as a reference upon which to center three overlapping docking grids (Figure 3a). Each window was then probed with fragments ultimately covering the whole putative interaction substructure. Interestingly, the docking analysis returned a characteristic result: only two fragments of interest docked proficiently in the 1–2 region, while all others docked in portion 3. It is worth noting that of the two fragments targeting region 3, one is characterized by the presence of a sulfonamide functionality, the same group as the one present in Venetoclax, an active Bcl-targeting FDA-approved drug. Importantly, Venetoclax is shown to engage the same region as the one we predicted here: it is worth underlining that no prior information on the Venetoclax-Bcl complex was used here.^31^ The two fragments in window 3 of the helix were then defined as the starting moieties onto which the different fragments selected for the other portions were connected with simple bridging groups (see Methods).

At the end of the process, we first calculated the Tanimoto similarity with Venetoclax (Figure 3b), and then, we docked the automatically designed molecules onto the MLCE-predicted region to rank-score possible hits (see the Supporting Information, Table S1). Importantly, the group of 30 molecules with the best docking scores contained a large number of molecules with a Tanimoto similarity with Venetoclax higher than 0.5 (Figure 3c). Furthermore, the poses and interactions with the receptor for the best scoring hits significantly trace the ones observed for the Venetoclax-Bcl complex in the Protein Data Bank (Figure 3d,e).

Our PPI-Prediction plus FBDD approach proves able to provide significant information for the development of novel ligands for difficult targets. Indeed, in the context of a blind design exercise, the strategy we have delineated can proficiently unveil simple, yet viable, molecular starting points for the development of PPI-targeting drugs.

### VHL

The von Hippel–Lindau (VHL) protein plays a key role as the tumor suppressor by forming protein complexes with other proteins within the cell.^57^ Indeed, the VHL gene encodes a protein that is involved in the ubiquitination and degradation of hypoxia-inducible-factor (HIF), a transcription factor with a key role in the regulation of gene expression by oxygen. Importantly, the VHL protein is the substrate receptor subunit of the (RING)-VHL (CRL2^VHL^) multi-subunit E3 ligase, an enzyme of the ligase family, essential for guiding intracellular protein degradation via the ubiquitin–proteasome system (UPS).^27^ VHL has been targeted with small molecules with the aims of disrupting its deranged interactions with HIFs and hijacking the protein to form complexes with non-native neo-substrate proteins using proteolysis targeting chimeras (PROTACs) and induce the UPS degradation of pathologic proteins. The discovery of VHL ligands entailed both HTS campaigns and rational design efforts initially based on mimicking the critical PPIs of the HIF-1α/VHL complex.^58^

**Figure 4:**
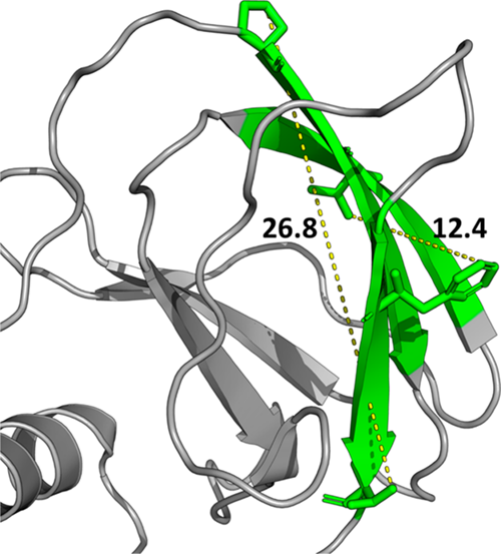
Development of HITs for VHL: the large surface of VHL. The largest surface targeted in this study is reported in this figure. The measures reported indicate the distances between extreme points of the surface.

**Figure 5:**
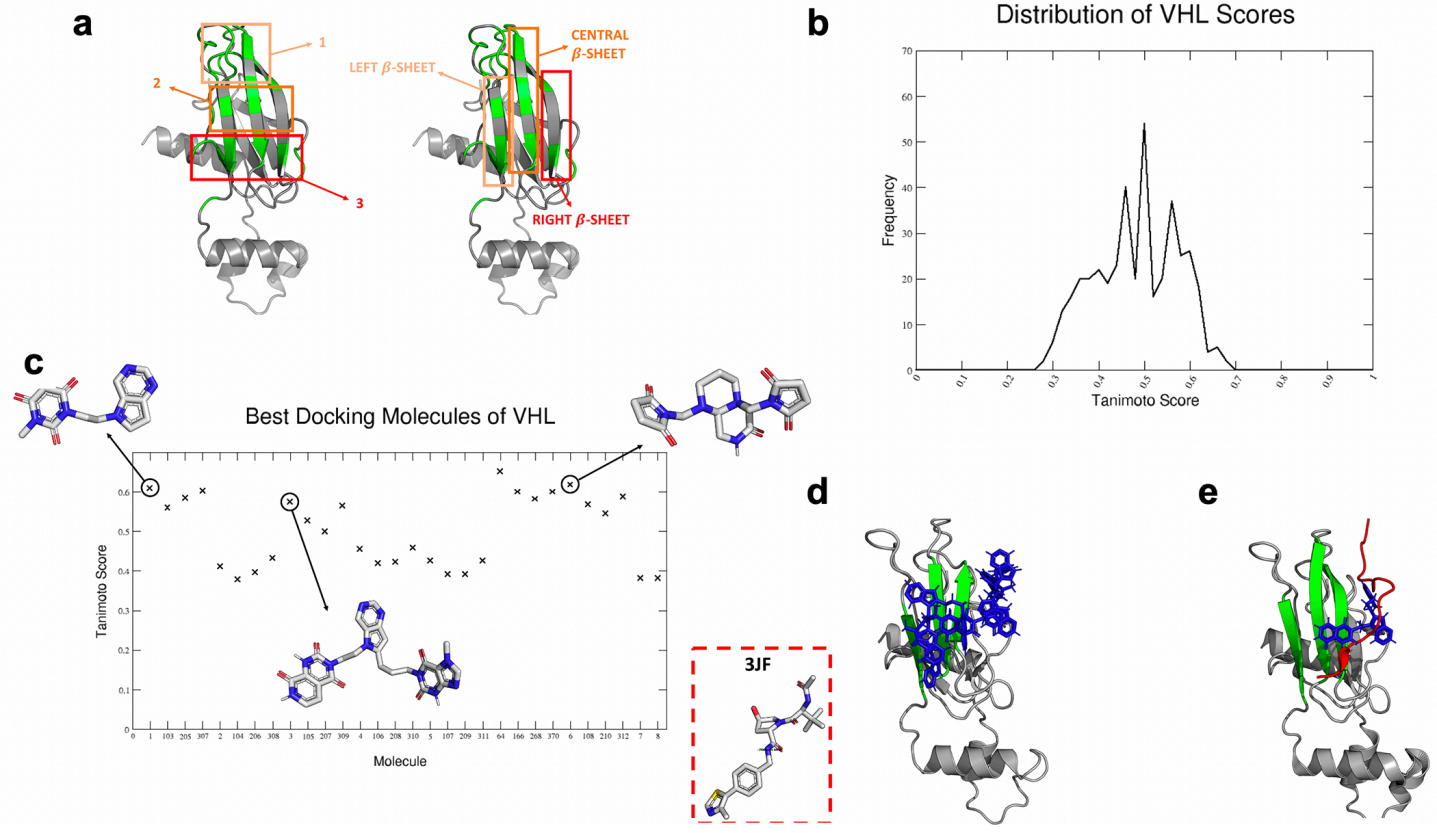
Development of HITs for VHL. (a) Docking sectors built for VHL based on the result of MLCE analysis. On the left, the three sectors encompassing the surface and targeted by fragment docking are oriented horizontally with respect to the main axis of the protein. On the right, the three docking sectors are oriented vertically and allow targeting β-sheets individually. (b) Distribution of the Tanimoto similarity scores, described in the main text, for all the VHL-binding HITs against experimentally determined VHL ligands (see the text). (c) Tanimoto scores of the 30 best molecules (reported on the *X*-axis) derived from final docking analysis. The three molecules shown are some examples of the ligands found with FOES. In the box, an experimentally characterized active ligand is shown (3JF: PDB code 4W9H). (d) The best docking poses on VHL of the five best ligands built with FOES. Focusing on the green areas, it is clear that the FOES-generated ligands engage most of the PPI surface. (e) Red color indicates the peptide co-crystallized with VHL, and blue color indicates one of the representatives of best docking generating HITs. Although the surface involved in the binding is very large, there is a significant overlap in binding for the designed and experimental ligands.

From the structural point of view, this protein turned out to be the most complex: MLCE analysis identified a large interaction surface spanning residues 14–19, 47–51, and 89–93. This predicted interaction area is composed of three β-sheets spanning approximately 27.0 Å in length and 12 Å in width (Figure 4). Considering the extension of the potential PPI area, six grids were considered for fragment docking, as exemplified in Figure 5a. Only fragments with a docking score of −3.5 or lower and interacting with at least one of the β-sheets were considered for further combination. The resulting designed molecules were then docked onto the target area, and the set of 30 ligands with the best docking scores were selected as possible starting HITs (see the Supporting Information, Tables S2–S5).

Importantly, the Tanimoto similarity score is calculated between the selected hits (first and second generation used to build PROTACs)^59^ and known ligands from PDB structures (PDB codes: 4B9K, 4W9H) (Figure 5b,c).

Overall, the best scoring designer hits can aptly represent a viable starting point for the development of drug-like molecules targeting a difficult, but important, PPI surface (Figure 5d,e).

### HIV Integrase

HIV integrase is one of the main pharmacological targets, along with reverse DNA polymerase and HIV protease, which are pursued in the search for anti-AIDS therapies. The protein catalyzes the entry of viral DNA into the host DNA. This protein consists of three domains: N-terminal, C-terminal, and the central binding domain.^60^

**Figure 6:**
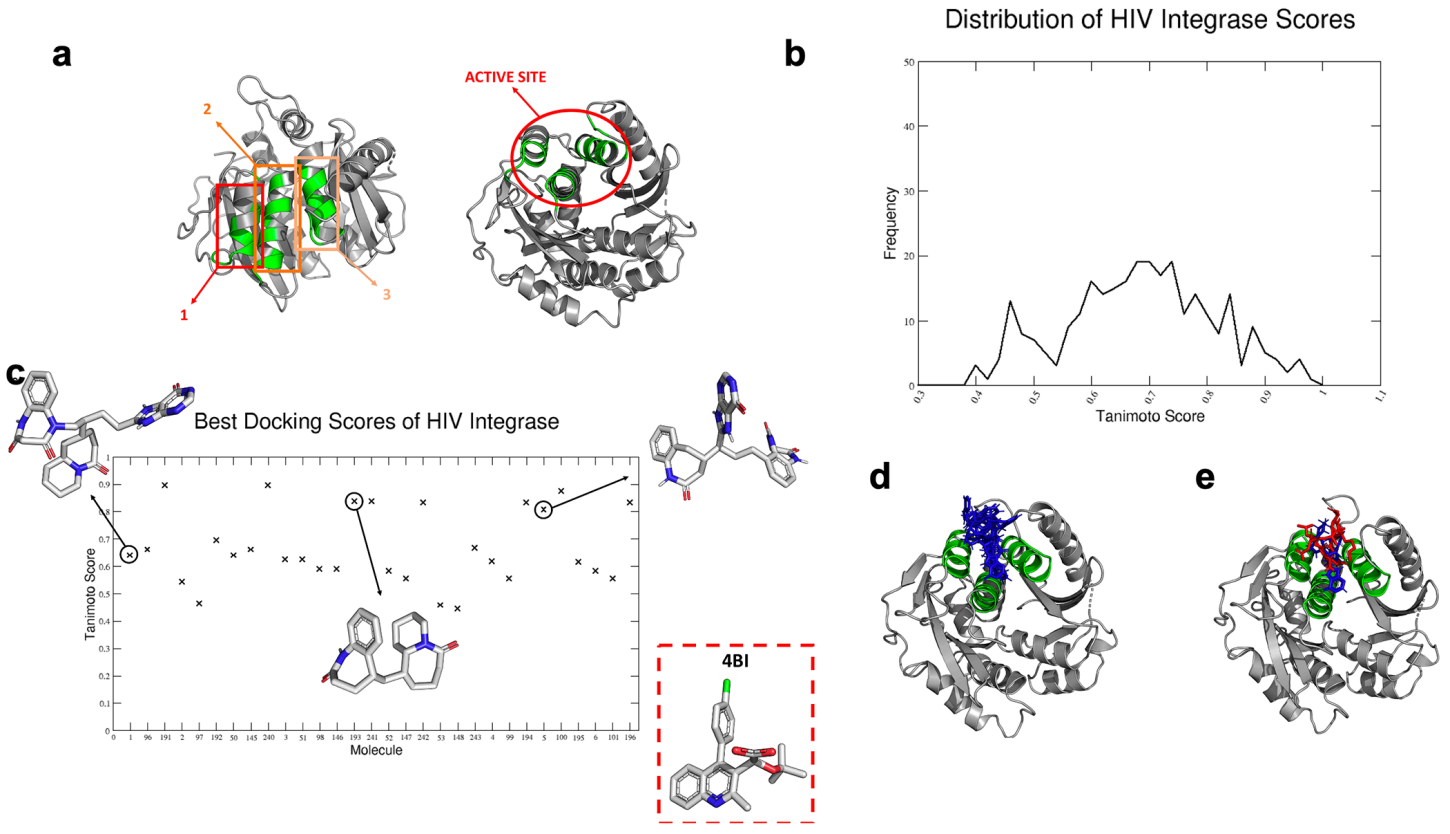
Development of HITs for HIV integrase. (a) Docking sectors built for HIV integrase based on the result of MLCE analysis. Interestingly, in this case, the predicted interaction area also encompasses the active site. (b) Distribution of the Tanimoto similarity scores, described in the main text, for all the HIV integrase-binding HITs generated against the experimentally characterized inhibitor. (c) Tanimoto score of the 30 best docking molecules (on the *X*-axis) derived from the final docking analysis. We show a few examples of molecules along the ranking. In the box, the experimental reference ligand (4BI, PDB code 4NYF) is shown. (d) Best docking poses on HIV integrase of the five best ligands built with FOES. Focusing on the green areas, it is clear that the FOES-generated ligands engage most of the PPI surface. (e) Red color indicates the ligand co-crystallized with HIV integrase, and blue color indicates one of the representatives of best docking generating HITs. Importantly, the two ligands engage the same portion of the protein.

First, MLCE analysis identified the three helices that constitute the binding site as a possible zone of interaction (Figure 6a). This binding site is composed of residues 38–50, 68–77, and 258–271 (PDB code: 3L3U). In contrast to the other examples discussed here, this site is well-defined and spatially confined. Importantly, considering the importance of the target, it has been extensively validated.^61^

To test our integrated PPI identification and FBDD approach, we first subdivided the predicted binding region in three overlapping docking windows as described above. Additionally, given the more compact nature of the interaction area, we examined the performance of using one single window spanning the three α-helices that enclose the predicted region of interest were considered (Figure 6a). In both cases, the fragments bind the PPI region with optimal docking scores (see the Supporting Information).

A challenging aspect of HIV integrase as a target is that known active ligands are highly drug like: given the nature of our design protocol, based on combining fragments that span potentially extended regions, emerging molecules could be expected to be large and flexible. Notably, the physicochemical characteristics of the designs matched well with those of experimentally tested ligands. Indeed, good similarity scores were obtained for all ligands tested (Figure 6b).

Importantly, the final docking analysis on the target site showed that, among the set with the top ranking docked hits, several designs showed a Tanimoto score significantly above 0.500 with experimental ligands (Figure 6c) (see the Supporting Information, Tables S6–S8).

These results further confirm our working hypothesis: it is possible to obtain starting HIT molecules that are very similar to already known ligands in a simple, intuitive, and unbiased manner. In the case of HIV integrase, it is clear that minor medicinal chemistry may be sufficient to make the HIT (Figure 6d,e) molecules more drug-like and ready to be tested by biological assays.

## Discussion

Herein, we have presented a simple model that demonstrates how interesting ligands of protein interaction surfaces can emerge on the basis of considerations of proteins’ structural and physical–chemistry properties. We have shown that the specific energetic signature of predicted PPI surfaces, integrated with information on the geometrical, hydrogen bonding, and steric constraints they place on potentially binding fragments, is sufficient to guide a viable selection of PPI-targeting ligands with interesting initial drug-like profiles. These ligands correspond to structures comprising the menu of functional groups and hydrophobic moieties found by typical drug design efforts.

Our results thus provide a general framework to favor the emergence and selection of the key characteristics necessary for a ligand to bind a PPI surface. The model we propose is consistent with the fact that protein interaction surfaces are selected by Nature with specific physico-chemical and structural properties to enable efficient recognition of partners.^3,39,62−64^ In this framework, the specific energetic organization of PPI surfaces for function can, on the one hand, be exploited for the prediction of the interaction region and, on the other hand, to define the sterics and functionalities necessary for proper binding to such areas.

PPI targeting has blossomed into a very broad and attractive field for drug discovery, as many pathologic processes depend on the PPI malfunction.^65^ However, a large number of proteins involved still remain undruggable due to the lack of well-defined binding sites and to the characteristics of the regions involved in interactions. The latter, being typically large and solvent-exposed, pose a real challenge to classic drug-discovery approaches. Indeed, while numerous methods (such as HTS, virtual screening, and fragments design) have been tested, success has been somewhat limited. In this context, the recent use of physics-based models that take the dynamics of the target protein explicitly into account combined with fragment-binding simulations has proven successful for the simultaneous discovery of cryptic sites and of suitable binding moieties. In this context, fragment-based approaches are interesting and attractive because they allow ad hoc construction of new ligands using the target as a template, directing the selection of functional groups based on the interactions that are presented by the protein to potential partners.^66^

Our goal in this work was to make the construction of new HITs as direct and simple as possible using an unbiased prediction of potential protein interaction surfaces. We demonstrated that it is possible to find “raw” starting molecules that, with classical medicinal chemistry modifications, could lead to novel leads. A possible caveat to mention here is that the hits, at this stage, are not evaluated or screened for their synthesizability: integrating this feature would aptly improve the quality of the selection and the prioritization of compounds. An important point worth noting here is that the designed molecules emerging from our work already show a high degree of similarity to known active ligands, a result that is particularly significant considering that no prior information on binding regions or ligand identities was used.^67^

This clearly supports the possibility of applying FOES to yet undruggable proteins or orphan targets. Thanks to the simple and straightforward nature of the method, the simple knowledge of the 3D structure of the protein is the only requirement to start the molecular design process. In this context, the limitation of possible fragment-docking areas helps make the selection of optimal fragments and the construction of new ligands effective. We suggest that our approach can find applicative venues in a wide range of problems.

## Conclusions

In summary, we have presented a novel, simple, and general design strategy that integrates the characterization of the dynamics and energetics signature of specific protein regions involved in interactions with other proteins with the docking, selection, and combination of drug-like fragments. This approach naturally permits us to identify the binding determinants of new PPI inhibitor candidates. This protocol was applied to study difficult and important pharmacologic targets whose cell mechanisms are indeed linked to the formation of PPIs, namely, Bcl, VHL, and HIV1 integrase. FOES allowed us to explore the molecular diversity space of potential ligands, with no requirement of prior information on the location and properties of potential interaction surface inhibitors or on the structures of potential lead compounds. Importantly, the ensembles of best designed candidates as PPI antagonists contain a significant number of HITs with a high chemical similarity to known active PPI inhibitors that had previously been experimentally tested. The ability to generate novel actionable compounds ab initio provides viable opportunities for the further lead development and refinement.

Finally, our results may have larger ramifications in the understanding of the mechanisms of chemical biology via the possibility to design ligands that modify functional interactions and demonstrate an unappreciated delicate interplay between the dynamics and energetics of specific protein regions and the possibility to exploit these traits for molecular design.

## Supporting Information Available

All data for docking scores and similarity scores (PDF)

## Notes

MLCE implementation is publicly available on GitHub at https://github.com/colombolab/MLCE. The docking, MD, and clustering algorithms used here are available as modules of the MAESTRO Suite of Programs.
