# Supplementary material for: Integrating Protein Interaction Surface Prediction with a Fragment-Based Drug Design: Automatic Design of New Leads with Fragments on Energy Surfaces
Source: J Chem Inf Model. 2022 Dec 27;63(1):343–53. doi: 10.1021/acs.jcim.2c01408 (PMC9832486; doi:10.1021/acs.jcim.2c01408)
Supplement: Supplementary file 1 — ci2c01408_si_001.pdf [file ci2c01408_si_001.pdf]

Supporting Information for:

Integrating Protein Interaction Surface Prediction With Fragment Based Drug Design: Automatic Design Of New Leads With FOES (Fragments On Energy Surfaces)

Luca Torielli,[1] Stefano A. Serapian,[1] Lara Mussolin,[2,3] Elisabetta Moroni,[4] Giorgio Colombo[1]

1. University of Pavia, Department of Chemistry, Via Taramelli 12 27100, Pavia, Italy
2. Department of Woman's and Child's Health, Pediatric Hematology, Oncology and Stem Cell Transplant Center, University of Padua, Via Giustiniani, 3 – 35128, Padua, Italy
3. Istituto di Ricerca Pediatrica Città della Speranza, Corso Stati Uniti, 4 F, 35127, Padova, Italy
4. SCITEC-CNR, via Mario Bianco 9, 20131, Milano, Italy

Author to whom correspondence should be addressed: Giorgio Colombo, g.colombo@unipv.it

The following tables report the scoring data for all the fragments found in the libraries downloadable from www.schrodinger.com

Table S1. Our hit compounds ranked by Docking score (XP GScore) with Tanimoto score based on the similarity with Venetoclax

Canvas Tanimoto Similarity	XP GScore	Fragment number
0.555	-8.728	1
0.507	-8.326	2
0.565	-8.270	3
0.555	-8.247	4
0.489	-8.224	5
0.500	-8.137	6
0.485	-8.089	7
0.522	-8.050	8
0.544	-7.998	9
0.544	-7.997	10
0.551	-7.973	11
0.500	-7.972	12
0.533	-7.971	13
0.474	-7.842	14
0.537	-7.833	15
0.529	-7.808	16
0.551	-7.808	17
0.485	-7.801	18
0.555	-7.794	19
0.529	-7.767	20
0.493	-7.766	21
0.485	-7.744	22
0.544	-7.743	23
0.493	-7.727	24
0.496	-7.722	25
0.565	-7.712	26
0.576	-7.711	27
0.504	-7.700	28
0.507	-7.634	29
0.489	-7.584	30
0.485	-7.556	31
0.529	-7.542	32
0.565	-7.515	33
0.485	-7.492	34
0.572	-7.471	35
0.561	-7.469	36
0.485	-7.466	37
0.571	-7.466	38
0.507	-7.457	39

0.493	-7.456	40
0.481	-7.444	41
0.522	-7.436	42
0.522	-7.435	43
0.555	-7.426	44
0.507	-7.387	45
0.485	-7.376	46
0.514	-7.353	47
0.511	-7.341	48
0.522	-7.340	49
0.544	-7.334	50
0.555	-7.328	51
0.489	-7.309	52
0.489	-7.309	53
0.580	-7.309	54
0.576	-7.299	55
0.252	-7.282	56
0.485	-7.274	57
0.562	-7.256	58
0.558	-7.256	59
0.496	-7.250	60
0.478	-7.246	61
0.485	-7.242	62
0.558	-7.217	63
0.485	-7.209	64
0.558	-7.206	65
0.555	-7.206	66
0.529	-7.188	67
0.485	-7.185	68
0.474	-7.130	69
0.571	-7.127	70
0.558	-7.126	71
0.561	-7.123	72
0.493	-7.118	73
0.529	-7.111	74
0.561	-7.098	75
0.536	-7.082	76
0.496	-7.072	77
0.540	-7.066	78
0.518	-7.051	79
0.561	-7.049	80
0.485	-7.042	81
0.493	-7.040	82
0.500	-7.037	83
0.481	-7.006	84
0.529	-6.991	85

0.565	-6.979	86
0.500	-6.979	87
0.518	-6.964	88
0.518	-6.956	89
0.544	-6.942	90
0.514	-6.941	91
0.547	-6.935	92
0.522	-6.929	93
0.540	-6.928	94
0.485	-6.920	95
0.522	-6.917	96
0.500	-6.915	97
0.481	-6.912	98
0.496	-6.907	99
0.489	-6.904	100
0.489	-6.891	101
0.514	-6.877	102
0.580	-6.869	103
0.485	-6.864	104
0.558	-6.851	105
0.522	-6.848	106
0.569	-6.848	107
0.496	-6.843	108
0.522	-6.836	109
0.507	-6.836	110
0.496	-6.817	111
0.474	-6.816	112
0.481	-6.809	113
0.481	-6.798	114
0.496	-6.796	115
0.533	-6.784	116
0.514	-6.780	117
0.550	-6.771	118
0.526	-6.756	119
0.496	-6.753	120
0.522	-6.720	121
0.537	-6.706	122
0.489	-6.686	123
0.561	-6.685	124
0.518	-6.685	125
0.522	-6.681	126
0.489	-6.677	127
0.571	-6.673	128
0.504	-6.672	129
0.555	-6.653	130
0.518	-6.644	131

0.558	-6.643	132
0.493	-6.623	133
0.514	-6.618	134
0.493	-6.614	135
0.569	-6.607	136
0.485	-6.602	137
0.514	-6.574	138
0.500	-6.567	139
0.504	-6.560	140
0.576	-6.552	141
0.485	-6.539	142
0.514	-6.535	143
0.514	-6.533	144
0.544	-6.530	145
0.504	-6.509	146
0.474	-6.487	147
0.565	-6.485	148
0.493	-6.482	149
0.504	-6.477	150
0.514	-6.453	151
0.489	-6.451	152
0.544	-6.449	153
0.518	-6.432	154
0.518	-6.432	155
0.507	-6.429	156
0.554	-6.426	157
0.522	-6.421	158
0.485	-6.419	159
0.500	-6.416	160
0.565	-6.412	161
0.496	-6.388	162
0.489	-6.387	163
0.522	-6.376	164
0.551	-6.374	165
0.540	-6.368	166
0.554	-6.367	167
0.485	-6.360	168
0.478	-6.358	169
0.485	-6.354	170
0.547	-6.353	171
0.318	-6.351	172
0.544	-6.350	173
0.562	-6.348	174
0.529	-6.345	175
0.518	-6.340	176
0.518	-6.331	177

0.568	-6.327	178
0.496	-6.326	179
0.529	-6.325	180
0.496	-6.323	181
0.500	-6.315	182
0.518	-6.311	183
0.504	-6.296	184
0.577	-6.291	185
0.518	-6.286	186
0.565	-6.276	187
0.565	-6.276	188
0.533	-6.263	189
0.555	-6.262	190
0.565	-6.247	191
0.504	-6.242	192
0.507	-6.239	193
0.561	-6.223	194
0.569	-6.222	195
0.569	-6.222	196
0.529	-6.218	197
0.485	-6.210	198
0.529	-6.207	199
0.529	-6.205	200
0.576	-6.199	201
0.511	-6.198	202
0.555	-6.195	203
0.522	-6.191	204
0.229	-6.189	205
0.485	-6.181	206
0.485	-6.175	207
0.522	-6.174	208
0.485	-6.171	209
0.485	-6.165	210
0.576	-6.159	211
0.518	-6.154	212
0.504	-6.150	213
0.214	-6.149	214
0.507	-6.125	215
0.522	-6.094	216
0.526	-6.093	217
0.504	-6.087	218
0.500	-6.087	219
0.533	-6.086	220
0.561	-6.084	221
0.485	-6.082	222
0.489	-6.081	223

0.540	-6.073	224
0.529	-6.064	225
0.551	-6.031	226
0.558	-6.030	227
0.554	-6.023	228
0.504	-6.021	229
0.565	-6.000	230
0.504	-5.993	231
0.526	-5.985	232
0.568	-5.985	233
0.568	-5.981	234
0.551	-5.962	235
0.558	-5.958	236
0.555	-5.958	237
0.493	-5.956	238
0.237	-5.952	239
0.500	-5.938	240
0.558	-5.938	241
0.485	-5.932	242
0.500	-5.920	243
0.555	-5.916	244
0.489	-5.911	245
0.522	-5.908	246
0.558	-5.904	247
0.544	-5.904	248
0.507	-5.899	249
0.514	-5.875	250
0.518	-5.838	251
0.496	-5.836	252
0.544	-5.829	253
0.526	-5.828	254
0.548	-5.799	255
0.290	-5.797	256
0.555	-5.789	257
0.237	-5.784	258
0.533	-5.775	259
0.551	-5.768	260
0.533	-5.766	261
0.533	-5.765	262
0.244	-5.747	263
0.558	-5.746	264
0.504	-5.738	265
0.489	-5.723	266
0.500	-5.693	267
0.540	-5.689	268
0.572	-5.676	269

0.576	-5.666	270
0.576	-5.666	271
0.493	-5.650	272
0.555	-5.628	273
0.485	-5.626	274
0.493	-5.625	275
0.489	-5.622	276
0.260	-5.620	277
0.481	-5.602	278
0.341	-5.601	279
0.584	-5.593	280
0.551	-5.591	281
0.518	-5.586	282
0.518	-5.584	283
0.507	-5.581	284
0.555	-5.557	285
0.558	-5.549	286
0.507	-5.549	287
0.504	-5.538	288
0.558	-5.531	289
0.522	-5.524	290
0.562	-5.518	291
0.565	-5.514	292
0.561	-5.505	293
0.507	-5.492	294
0.561	-5.485	295
0.522	-5.483	296
0.504	-5.467	297
0.580	-5.464	298
0.303	-5.461	299
0.441	-5.457	300
0.507	-5.450	301
0.522	-5.425	302
0.540	-5.416	303
0.485	-5.412	304
0.540	-5.410	305
0.485	-5.396	306
0.576	-5.395	307
0.478	-5.392	308
0.537	-5.381	309
0.481	-5.378	310
0.485	-5.371	311
0.485	-5.359	312
0.485	-5.354	313
0.518	-5.347	314
0.496	-5.325	315

0.555	-5.324	316
0.565	-5.323	317
0.533	-5.320	318
0.544	-5.319	319
0.558	-5.309	320
0.474	-5.306	321
0.569	-5.300	322
0.533	-5.289	323
0.580	-5.265	324
0.555	-5.264	325
0.555	-5.263	326
0.500	-5.246	327
0.496	-5.244	328
0.561	-5.219	329
0.518	-5.215	330
0.368	-5.214	331
0.526	-5.213	332
0.229	-5.208	333
0.529	-5.197	334
0.558	-5.192	335
0.293	-5.183	336
0.555	-5.181	337
0.576	-5.175	338
0.489	-5.147	339
0.489	-5.127	340
0.568	-5.124	341
0.533	-5.116	342
0.537	-5.102	343
0.353	-5.094	344
0.540	-5.093	345
0.576	-5.089	346
0.518	-5.089	347
0.565	-5.075	348
0.551	-5.075	349
0.580	-5.068	350
0.522	-5.067	351
0.481	-5.052	352
0.478	-5.047	353
0.529	-5.040	354
0.565	-5.039	355
0.485	-5.038	356
0.518	-5.036	357
0.308	-5.035	358
0.221	-5.032	359
0.561	-5.017	360
0.485	-5.017	361

0.229	-5.015	362
0.353	-5.038	363
0.562	-5.010	364
0.558	-5.007	365
0.507	-5.005	366
0.551	-5.005	367
0.551	-4.998	368
0.326	-4.985	369
0.518	-4.977	370
0.518	-4.977	371
0.500	-4.951	372
0.326	-4.938	373
0.555	-4.933	374
0.504	-4.929	375
0.576	-4.929	376
0.555	-4.918	377
0.493	-4.912	378
0.500	-4.908	379
0.323	-4.914	380
0.485	-4.895	381
0.214	-4.892	382
0.518	-4.877	383
0.526	-4.874	384
0.576	-4.871	385
0.507	-4.849	386
0.496	-4.831	387
0.555	-4.825	388
0.562	-4.822	389
0.301	-4.776	390
0.551	-4.773	391
0.478	-4.769	392
0.551	-4.769	393
0.580	-4.766	394
0.500	-4.766	395
0.493	-4.752	396
0.267	-4.748	397
0.558	-4.745	398
0.288	-4.750	399
0.558	-4.741	400
0.252	-4.739	401
0.514	-4.739	402
0.555	-4.736	403
0.555	-4.736	404
0.533	-4.736	405
0.558	-4.715	406
0.547	-4.715	407

0.561	-4.700	408
0.280	-4.699	409
0.558	-4.691	410
0.331	-4.682	411
0.485	-4.672	412
0.558	-4.664	413
0.323	-4.661	414
0.558	-4.649	415
0.504	-4.632	416
0.572	-4.628	417
0.555	-4.622	418
0.478	-4.620	419
0.555	-4.608	420
0.518	-4.605	421
0.449	-4.604	422
0.474	-4.604	423
0.422	-4.604	424
0.295	-4.609	425
0.580	-4.600	426
0.551	-4.597	427
0.558	-4.593	428
0.235	-4.576	429
0.311	-4.595	430
0.474	-4.563	431
0.260	-4.560	432
0.364	-4.558	433
0.293	-4.555	434
0.522	-4.554	435
0.214	-4.552	436
0.572	-4.543	437
0.518	-4.536	438
0.562	-4.536	439
0.481	-4.535	440
0.562	-4.517	441
0.562	-4.513	442
0.323	-4.512	443
0.551	-4.499	444
0.548	-4.497	445
0.191	-4.480	446
0.551	-4.470	447
0.529	-4.467	448
0.559	-4.462	449
0.504	-4.459	450
0.252	-4.452	451
0.558	-4.448	452
0.344	-4.444	453

0.555	-4.443	454
0.511	-4.442	455
0.558	-4.424	456
0.507	-4.420	457
0.576	-4.417	458
0.500	-4.398	459
0.391	-4.395	460
0.474	-4.395	461
0.529	-4.392	462
0.555	-4.387	463
0.507	-4.375	464
0.500	-4.371	465
0.361	-4.371	466
0.489	-4.370	467
0.576	-4.368	468
0.331	-4.362	469
0.493	-4.358	470
0.555	-4.352	471
0.558	-4.351	472
0.500	-4.345	473
0.551	-4.338	474
0.518	-4.335	475
0.326	-4.314	476
0.244	-4.313	477
0.456	-4.303	478
0.580	-4.301	479
0.338	-4.299	480
0.333	-4.298	481
0.540	-4.284	482
0.229	-4.269	483
0.485	-4.265	484
0.555	-4.262	485
0.533	-4.246	486
0.441	-4.246	487
0.504	-4.244	488
0.391	-4.236	489
0.526	-4.234	490
0.529	-4.232	491
0.572	-4.216	492
0.331	-4.214	493
0.456	-4.203	494
0.333	-4.211	495
0.558	-4.198	496
0.526	-4.198	497
0.514	-4.189	498
0.561	-4.187	499

0.420	-4.184	500
0.561	-4.182	501
0.250	-4.182	502
0.529	-4.161	503
0.229	-4.153	504
0.328	-4.152	505
0.500	-4.150	506
0.507	-4.150	507
0.260	-4.128	508
0.290	-4.126	509
0.537	-4.125	510
0.321	-4.112	511
0.551	-4.103	512
0.407	-4.100	513
0.558	-4.094	514
0.558	-4.094	515
0.562	-4.086	516
0.368	-4.086	517
0.507	-4.083	518
0.280	-4.081	519
0.533	-4.070	520
0.568	-4.052	521
0.522	-4.050	522
0.568	-4.041	523
0.237	-4.035	524
0.529	-4.030	525
0.518	-4.027	526
0.485	-3.997	527
0.353	-4.016	528
0.361	-4.006	529
0.440	-3.963	530
0.221	-3.962	531
0.551	-3.961	532
0.568	-3.956	533
0.529	-3.948	534
0.529	-3.948	535
0.326	-3.945	536
0.555	-3.938	537
0.456	-3.935	538
0.391	-3.957	539
0.308	-3.920	540
0.237	-3.919	541
0.321	-3.942	542
0.449	-3.915	543
0.338	-3.914	544
0.353	-3.913	545

0.548	-3.912	546
0.376	-3.903	547
0.489	-3.894	548
0.558	-3.892	549
0.351	-3.890	550
0.244	-3.888	551
0.328	-3.879	552
0.544	-3.878	553
0.409	-3.854	554
0.496	-3.851	555
0.529	-3.849	556
0.336	-3.847	557
0.496	-3.843	558
0.496	-3.843	559
0.351	-3.840	560
0.504	-3.836	561
0.326	-3.834	562
0.507	-3.812	563
0.326	-3.802	564
0.311	-3.808	565
0.507	-3.796	566
0.507	-3.796	567
0.258	-3.794	568
0.336	-3.792	569
0.504	-3.790	570
0.504	-3.790	571
0.568	-3.777	572
0.214	-3.774	573
0.565	-3.768	574
0.569	-3.768	575
0.561	-3.759	576
0.493	-3.749	577
0.551	-3.748	578
0.489	-3.735	579
0.452	-3.730	580
0.331	-3.730	581
0.282	-3.718	582
0.555	-3.716	583
0.353	-3.713	584
0.346	-3.696	585
0.463	-3.694	586
0.393	-3.694	587
0.331	-3.682	588
0.229	-3.679	589
0.444	-3.678	590
0.378	-3.677	591

0.500	-3.677	592
0.565	-3.673	593
0.456	-3.666	594
0.361	-3.663	595
0.206	-3.658	596
0.258	-3.657	597
0.364	-3.656	598
0.540	-3.646	599
0.303	-3.638	600
0.551	-3.635	601
0.371	-3.642	602
0.485	-3.629	603
0.336	-3.626	604
0.555	-3.626	605
0.485	-3.610	606
0.562	-3.607	607
0.485	-3.602	608
0.444	-3.598	609
0.493	-3.594	610
0.229	-3.568	611
0.419	-3.567	612
0.500	-3.561	613
0.341	-3.561	614
0.500	-3.543	615
0.500	-3.534	616
0.303	-3.534	617
0.301	-3.533	618
0.522	-3.529	619
0.391	-3.546	620
0.326	-3.518	621
0.514	-3.513	622
0.260	-3.495	623
0.504	-3.495	624
0.237	-3.492	625
0.428	-3.491	626
0.448	-3.486	627
0.474	-3.484	628
0.415	-3.463	629
0.391	-3.462	630
0.460	-3.423	631
0.308	-3.423	632
0.493	-3.423	633
0.293	-3.418	634
0.338	-3.418	635
0.331	-3.442	636
0.434	-3.409	637

0.463	-3.405	638
0.431	-3.400	639
0.489	-3.398	640
0.353	-3.389	641
0.183	-3.384	642
0.346	-3.383	643
0.237	-3.379	644
0.422	-3.373	645
0.485	-3.369	646
0.338	-3.365	647
0.418	-3.365	648
0.353	-3.365	649
0.288	-3.362	650
0.459	-3.343	651
0.500	-3.329	652
0.237	-3.303	653
0.235	-3.302	654
0.456	-3.301	655
0.363	-3.296	656
0.252	-3.295	657
0.323	-3.289	658
0.518	-3.288	659
0.507	-3.287	660
0.311	-3.308	661
0.555	-3.282	662
0.353	-3.280	663
0.351	-3.270	664
0.526	-3.267	665
0.504	-3.267	666
0.333	-3.266	667
0.500	-3.265	668
0.504	-3.263	669
0.507	-3.253	670
0.467	-3.252	671
0.504	-3.248	672
0.500	-3.246	673
0.221	-3.236	674
0.485	-3.228	675
0.507	-3.227	676
0.485	-3.225	677
0.489	-3.225	678
0.214	-3.222	679
0.481	-3.212	680
0.353	-3.223	681
0.456	-3.196	682
0.280	-3.201	683

0.353	-3.188	684
0.511	-3.186	685
0.496	-3.180	686
0.551	-3.179	687
0.338	-3.177	688
0.522	-3.174	689
0.353	-3.172	690
0.331	-3.171	691
0.328	-3.195	692
0.569	-3.151	693
0.551	-3.150	694
0.493	-3.147	695
0.568	-3.146	696
0.252	-3.146	697
0.331	-3.141	698
0.338	-3.133	699
0.444	-3.125	700
0.522	-3.119	701
0.313	-3.114	702
0.321	-3.103	703
0.282	-3.087	704
0.316	-3.078	705
0.343	-3.073	706
0.551	-3.064	707
0.555	-3.064	708
0.419	-3.060	709
0.507	-3.055	710
0.568	-3.053	711
0.434	-3.048	712
0.555	-3.046	713
0.425	-3.043	714
0.519	-3.036	715
0.406	-3.031	716
0.463	-3.031	717
0.449	-3.027	718
0.555	-3.026	719
0.569	-3.023	720
0.522	-3.018	721
0.295	-3.001	722
0.444	-2.992	723
0.507	-2.979	724
0.500	-2.969	725
0.214	-2.968	726
0.467	-2.963	727
0.430	-2.960	728
0.558	-2.959	729

0.303	-2.963	730
0.514	-2.928	731
0.444	-2.922	732
0.540	-2.912	733
0.540	-2.912	734
0.504	-2.908	735
0.338	-2.930	736
0.489	-2.895	737
0.250	-2.891	738
0.378	-2.887	739
0.333	-2.872	740
0.391	-2.868	741
0.507	-2.853	742
0.518	-2.853	743
0.353	-2.851	744
0.385	-2.844	745
0.456	-2.838	746
0.551	-2.833	747
0.459	-2.823	748
0.348	-2.823	749
0.361	-2.820	750
0.504	-2.808	751
0.318	-2.793	752
0.493	-2.789	753
0.467	-2.768	754
0.221	-2.764	755
0.353	-2.755	756
0.323	-2.745	757
0.311	-2.750	758
0.338	-2.741	759
0.431	-2.740	760
0.511	-2.729	761
0.323	-2.718	762
0.493	-2.711	763
0.370	-2.706	764
0.326	-2.663	765
0.323	-2.654	766
0.474	-2.643	767
0.341	-2.666	768
0.514	-2.638	769
0.526	-2.635	770
0.444	-2.630	771
0.338	-2.652	772
0.544	-2.622	773
0.252	-2.622	774
0.489	-2.608	775

0.493	-2.589	776
0.529	-2.586	777
0.348	-2.585	778
0.221	-2.567	779
0.459	-2.530	780
0.526	-2.511	781
0.419	-2.510	782
0.288	-2.510	783
0.449	-2.501	784
0.303	-2.520	785
0.323	-2.483	786
0.559	-2.473	787
0.418	-2.426	788
0.361	-2.389	789
0.430	-2.373	790
0.364	-2.368	791
0.540	-2.359	792
0.555	-2.358	793
0.418	-2.348	794
0.333	-2.344	795
0.385	-2.327	796
0.555	-2.321	797
0.453	-2.304	798
0.444	-2.291	799
0.370	-2.287	800
0.415	-2.250	801
0.375	-2.222	802
0.380	-2.216	803
0.333	-2.202	804
0.500	-2.199	805
0.580	-2.195	806
0.407	-2.191	807
0.485	-2.170	808
0.385	-2.170	809
0.522	-2.163	810
0.370	-2.146	811
0.380	-2.123	812
0.385	-2.119	813
0.415	-2.116	814
0.328	-2.111	815
0.565	-2.098	816
0.551	-2.093	817
0.544	-2.068	818
0.429	-2.060	819
0.580	-2.054	820
0.333	-2.058	821

0.551	-2.042	822
0.415	-2.020	823
0.504	-2.016	824
0.346	-2.012	825
0.341	-1.987	826
0.316	-1.979	827
0.540	-1.959	828
0.511	-1.956	829
0.518	-1.953	830
0.493	-1.924	831
0.370	-1.921	832
0.415	-1.909	833
0.526	-1.906	834
0.540	-1.906	835
0.183	-1.900	836
0.387	-1.899	837
0.568	-1.877	838
0.387	-1.875	839
0.430	-1.853	840
0.221	-1.833	841
0.551	-1.822	842
0.356	-1.815	843
0.561	-1.806	844
0.555	-1.800	845
0.214	-1.755	846
0.440	-1.742	847
0.489	-1.723	848
0.514	-1.703	849
0.514	-1.703	850
0.533	-1.671	851
0.558	-1.661	852
0.363	-1.647	853
0.555	-1.646	854
0.551	-1.621	855
0.576	-1.610	856
0.522	-1.518	857
0.514	-1.446	858
0.303	-1.431	859
0.558	-1.430	860
0.426	-1.380	861
0.363	-1.374	862
0.363	-1.165	863
0.543	-1.107	864
0.554	-1.106	865
0.533	-1.093	866
0.435	-0.943	867

0.554	-0.934	868
0.426	-0.928	869
0.547	-0.829	870
0.415	-0.775	871
0.580	-0.593	872
0.540	-0.582	873
0.544	-0.541	874
0.558	-0.496	875
0.518	-0.305	876
0.514	-0.262	877
0.206	-0.241	878
0.558	-0.213	879
0.518	0.063	880
0.518	0.090	881
0.518	0.122	882
0.576	0.260	883
0.514	0.386	884
0.551	0.484	885
0.580	0.593	886
0.576	0.666	887
0.514	0.782	888
0.551	0.939	889
0.526	0.988	890
0.555	1.048	891
0.565	1.075	892
0.555	1.094	893
0.529	1.367	894
0.514	1.526	895
0.580	1.826	896
0.565	2.765	897
0.493	3.667	898

Table S2. Our hit compounds ranked by Docking score (XP GScore) with Tanimoto score based on the similarity with PDB 4W9H

Canvas Tanimoto Similarity	XP GScore	Fragment number
0.609	-4.670	1
0.412	-4.033	2
0.588	-4.040	3
0.574	-4.027	4
0.485	-5.563	5
0.456	-3.740	6
0.426	-3.512	7
0.618	-3.369	8
0.382	-3.328	9
0.382	-3.328	10
0.500	-3.301	11
0.456	-3.217	12
0.544	-3.097	13
0.620	-3.064	14
0.588	-3.058	15
0.594	-3.115	16
0.629	-3.009	17
0.485	-2.970	18
0.648	-2.946	19
0.441	-2.903	20
0.441	-2.891	21
0.368	-2.959	22
0.338	-2.875	23
0.500	-2.874	24
0.529	-2.858	25
0.662	-2.852	26
0.456	-2.836	27
0.544	-2.903	28
0.574	-2.815	29
0.382	-2.811	30
0.614	-2.783	31
0.426	-2.741	32
0.574	-2.724	33
0.515	-2.715	34
0.353	-2.789	35
0.515	-2.708	36
0.544	-2.658	37
0.559	-2.652	38
0.544	-2.638	39
0.662	-2.641	40
0.485	-2.618	41
0.500	-2.612	42
0.603	-2.581	43

0.338	-2.555	44
0.338	-2.552	45
0.485	-2.548	46
0.618	-2.526	47
0.544	-2.525	48
0.605	-2.510	49
0.588	-2.508	50
0.544	-2.496	51
0.441	-2.461	52
0.441	-2.461	53
0.603	-2.444	54
0.485	-2.375	55
0.515	-2.365	56
0.485	-2.318	57
0.559	-2.317	58
0.500	-2.275	59
0.529	-2.275	60
0.500	-2.275	61
0.426	-2.270	62
0.544	-2.235	63
0.412	-2.206	64
0.629	-2.189	65
0.309	-2.240	66
0.515	-2.162	67
0.559	-2.149	68
0.485	-2.138	69
0.456	-2.128	70
0.426	-2.093	71
0.456	-2.085	72
0.515	-2.052	73
0.574	-2.034	74
0.529	-2.023	75
0.500	-2.021	76
0.368	-1.981	77
0.652	-1.973	78
0.456	-1.965	79
0.588	-1.948	80
0.544	-1.914	81
0.652	-3.495	82
0.536	-1.933	83
0.324	-3.482	84
0.456	-1.789	85
0.529	-1.737	86
0.397	-1.724	87
0.485	-1.689	88
0.382	-1.659	89

0.485	-1.638	90
0.559	-1.596	91
0.603	-3.201	92
0.309	-1.521	93
0.614	-3.095	94
0.647	-1.485	95
0.500	-1.372	96
0.397	-1.363	97
0.592	-2.983	98
0.338	-1.350	99
0.618	-1.299	100
0.471	-2.916	101
0.397	-1.256	102
0.426	-2.849	103
0.600	-1.101	104
0.529	-2.583	105
0.580	-2.411	106
0.324	-2.317	107
0.294	-2.229	108
0.353	-2.174	109
0.368	-2.150	110
0.353	-2.137	111
0.618	-2.130	112
0.368	-2.113	113
0.623	-2.070	114
0.382	-1.887	115
0.412	-1.824	116
0.353	-1.813	117
0.353	-1.766	118
0.353	-1.741	119
0.565	-1.734	120
0.426	-0.107	121
0.456	-1.428	122
0.620	-1.270	123
0.544	-0.568	124
0.632	2.402	125

Table S3. Our hit compounds ranked by Docking score (XP GScore) with Tanimoto score based on the similarity with Compound 130 in Bricelj, A.; Steinebach, C.; Kuchta, R.; Gütschow, M.; Sosič, I. E3 Ligase Ligands in Successful Protacs: An Overview of Syntheses and Linker Attachment Points. *Frontiers in Chemistry* **2021**, *9*, 707317

Canvas Tanimoto Similarity	XP GScore	Fragment number
0.560	-4.670	103
0.378	-4.033	104
0.527	-4.027	105
0.419	-3.740	106
0.392	-3.512	107
0.568	-3.369	108
0.351	-3.328	109
0.351	-3.328	110
0.459	-3.301	111
0.500	-3.097	112
0.541	-3.058	113
0.579	-3.009	114
0.618	-2.946	115
0.608	-2.852	116
0.419	-2.836	117
0.500	-2.903	118
0.527	-2.815	119
0.351	-2.811	120
0.566	-2.783	121
0.392	-2.741	122
0.527	-2.724	123
0.473	-2.715	124
0.473	-2.708	125
0.500	-2.658	126
0.514	-2.652	127
0.500	-2.638	128
0.608	-2.641	129
0.446	-2.618	130
0.459	-2.612	131
0.311	-2.555	132
0.311	-2.552	133
0.446	-2.548	134
0.500	-2.525	135
0.641	-2.510	136
0.541	-2.508	137
0.500	-2.496	138
0.405	-2.461	139
0.405	-2.461	140
0.554	-2.444	141
0.446	-2.375	142
0.473	-2.365	143
0.446	-2.318	144

0.514	-2.317	145
0.459	-2.275	146
0.459	-2.275	147
0.392	-2.270	148
0.500	-2.235	149
0.378	-2.206	150
0.579	-2.189	151
0.284	-2.240	152
0.473	-2.162	153
0.514	-2.149	154
0.446	-2.138	155
0.419	-2.128	156
0.419	-2.085	157
0.473	-2.052	158
0.527	-2.034	159
0.486	-2.023	160
0.459	-2.021	161
0.338	-1.981	162
0.600	-1.973	163
0.419	-1.965	164
0.500	-1.914	165
0.600	-3.495	166
0.493	-1.933	167
0.419	-1.789	168
0.486	-1.737	169
0.365	-1.724	170
0.446	-1.689	171
0.351	-1.659	172
0.446	-1.638	173
0.514	-1.596	174
0.554	-3.201	175
0.284	-1.521	176
0.587	-3.095	177
0.595	-1.485	178
0.459	-1.372	179
0.365	-1.363	180
0.311	-1.350	181
0.568	-1.299	182
0.432	-2.916	183
0.365	-1.256	184
0.392	-2.849	185
0.553	-1.101	186
0.486	-2.583	187
0.533	-2.411	188
0.297	-2.317	189
0.270	-2.229	190

0.324	-2.174	191
0.338	-2.150	192
0.324	-2.137	193
0.573	-2.070	194
0.351	-1.887	195
0.378	-1.824	196
0.324	-1.813	197
0.324	-1.766	198
0.324	-1.741	199
0.392	-0.107	200
0.419	-1.428	201
0.592	-1.270	202
0.500	-0.568	203
0.581	2.402	204

Table S4. Our hit compounds ranked by Docking score (XP GScore) with Tanimoto score based on the similarity with PDB 4B9K

Canvas Tanimoto Similarity	XP GScore	Fragment number
0.585	-4.670	205
0.397	-4.033	206
0.500	-4.027	207
0.422	-3.740	208
0.391	-3.512	209
0.545	-3.369	210
0.365	-3.328	211
0.365	-3.328	212
0.446	-3.301	213
0.516	-3.097	214
0.515	-3.058	215
0.559	-3.009	216
0.580	-2.946	217
0.591	-2.852	218
0.422	-2.836	219
0.470	-2.903	220
0.547	-2.815	221
0.344	-2.811	222
0.544	-2.783	223
0.391	-2.741	224
0.500	-2.724	225
0.462	-2.715	226
0.462	-2.708	227
0.516	-2.658	228
0.485	-2.652	229
0.492	-2.638	230
0.591	-2.641	231
0.431	-2.618	232
0.469	-2.612	233
0.317	-2.555	234
0.339	-2.552	235
0.431	-2.548	236
0.470	-2.525	237
0.541	-2.510	238
0.515	-2.508	239
0.492	-2.496	240
0.406	-2.461	241
0.406	-2.461	242
0.530	-2.444	243
0.453	-2.375	244
0.462	-2.365	245
0.453	-2.318	246

0.508	-2.317	247
0.446	-2.275	248
0.446	-2.275	249
0.435	-2.270	250
0.492	-2.235	251
0.397	-2.206	252
0.559	-2.189	253
0.286	-2.240	254
0.462	-2.162	255
0.485	-2.149	256
0.453	-2.138	257
0.422	-2.128	258
0.422	-2.085	259
0.462	-2.052	260
0.500	-2.034	261
0.500	-2.023	262
0.446	-2.021	263
0.328	-1.981	264
0.582	-1.973	265
0.400	-1.965	266
0.492	-1.914	267
0.582	-3.495	268
0.531	-1.933	269
0.422	-1.789	270
0.500	-1.737	271
0.381	-1.724	272
0.453	-1.689	273
0.344	-1.659	274
0.476	-1.638	275
0.485	-1.596	276
0.530	-3.201	277
0.306	-1.521	278
0.567	-3.095	279
0.576	-1.485	280
0.446	-1.372	281
0.426	-1.363	282
0.317	-1.350	283
0.545	-1.299	284
0.460	-2.916	285
0.426	-1.256	286
0.391	-2.849	287
0.576	-1.101	288
0.477	-2.583	289
0.554	-2.411	290
0.302	-2.317	291
0.270	-2.229	292

0.355	-2.174	293
0.328	-2.150	294
0.355	-2.137	295
0.552	-2.070	296
0.344	-1.887	297
0.375	-1.824	298
0.312	-1.813	299
0.312	-1.766	300
0.355	-1.741	301
0.413	-0.107	302
0.422	-1.428	303
0.574	-1.270	304
0.492	-0.568	305
0.561	2.402	306

Table S5. Our hit compounds ranked by Docking score (XP GScore) with Tanimoto score based on the similarity with Compound 107 present in Bricelj, A.; Steinebach, C.; Kuchta, R.; Gütschow, M.; Sosič, I. E3 Ligase Ligands in Successful Protacs: An Overview of Syntheses and Linker Attachment Points. *Frontiers in Chemistry* **2021**, *9*, 707317

Canvas Tanimoto Similarity	XP GScore	Fragment number
0.603	-4.670	307
0.433	-4.033	308
0.565	-4.027	309
0.459	-3.740	310
0.426	-3.512	311
0.587	-3.369	312
0.377	-3.328	313
0.377	-3.328	314
0.484	-3.301	315
0.557	-3.097	316
0.581	-3.058	317
0.600	-3.009	318
0.574	-2.946	319
0.609	-2.852	320
0.459	-2.836	321
0.532	-2.903	322
0.590	-2.815	323
0.377	-2.811	324
0.585	-2.783	325
0.403	-2.741	326
0.565	-2.724	327
0.500	-2.715	328
0.500	-2.708	329
0.557	-2.658	330
0.548	-2.652	331

0.532	-2.638	332
0.609	-2.641	333
0.468	-2.618	334
0.508	-2.612	335
0.328	-2.555	336
0.350	-2.552	337
0.468	-2.548	338
0.532	-2.525	339
0.534	-2.510	340
0.581	-2.508	341
0.532	-2.496	342
0.419	-2.461	343
0.419	-2.461	344
0.597	-2.444	345
0.492	-2.375	346
0.500	-2.365	347
0.492	-2.318	348
0.548	-2.317	349
0.484	-2.275	350
0.484	-2.275	351
0.475	-2.270	352
0.532	-2.235	353
0.433	-2.206	354
0.600	-2.189	355
0.317	-2.240	356
0.500	-2.162	357
0.548	-2.149	358
0.468	-2.138	359
0.459	-2.128	360
0.459	-2.085	361
0.500	-2.052	362
0.565	-2.034	363
0.541	-2.023	364
0.484	-2.021	365
0.361	-1.981	366
0.600	-1.973	367
0.435	-1.965	368
0.532	-1.914	369
0.600	-3.495	370
0.548	-1.933	371
0.459	-1.789	372
0.541	-1.737	373
0.393	-1.724	374
0.492	-1.689	375
0.377	-1.659	376
0.492	-1.638	377

0.548	-1.596	378
0.571	-3.201	379
0.317	-1.521	380
0.561	-3.095	381
0.619	-1.485	382
0.484	-1.372	383
0.441	-1.363	384
0.350	-1.350	385
0.613	-1.299	386
0.500	-2.916	387
0.441	-1.256	388
0.426	-2.849	389
0.594	-1.101	390
0.516	-2.583	391
0.571	-2.411	392
0.333	-2.317	393
0.300	-2.229	394
0.390	-2.174	395
0.361	-2.150	396
0.367	-2.137	397
0.594	-2.070	398
0.377	-1.887	399
0.410	-1.824	400
0.344	-1.813	401
0.344	-1.766	402
0.367	-1.741	403
0.426	-0.107	404
0.459	-1.428	405
0.567	-1.270	406
0.532	-0.568	407
0.603	2.402	408

Table S6. Our hit compounds ranked by Docking score (XP GScore) with Tanimoto score based on the similarity with PDB 4CEA

Canvas Tanimoto Similarity	XP GScore	Fragment number
0.640	-5.546	1
0.543	-4.834	2
0.625	-4.419	3
0.617	-4.269	4
0.808	-4.228	5
0.583	-4.193	6
0.723	-4.121	7
0.660	-4.012	8
0.638	-3.864	9
0.617	-3.848	10
0.574	-3.784	11
0.458	-3.638	12
0.646	-3.526	13
0.787	-3.524	14
0.638	-3.511	15
0.765	-3.510	16
0.686	-3.443	17
0.457	-3.250	18
0.604	-3.221	19
0.587	-3.217	20
0.543	-3.181	21
0.800	-3.161	22
0.702	-3.142	23
0.458	-3.131	24
0.574	-3.089	25
0.712	-3.014	26
0.511	-2.966	27
0.479	-2.931	28
0.673	-2.930	29
0.468	-2.929	30
0.532	-2.878	31
0.660	-2.835	32
0.729	-2.782	33
0.604	-2.713	34
0.780	-2.676	35
0.458	-2.667	36
0.562	-2.645	37
0.720	-2.624	38
0.712	-2.623	39
0.620	-2.455	40
0.714	-2.442	41

0.673	-2.405	42
0.592	-2.382	43
0.681	-2.367	44
0.653	-2.302	45
0.740	-2.222	46
0.489	-1.964	47
0.750	-1.820	48
0.776	-1.302	49
0.640	-4.761	50
0.625	-4.419	51
0.583	-4.390	52
0.458	-4.377	53
0.729	-4.110	54
0.583	-4.058	55
0.660	-4.012	56
0.800	-3.801	57
0.625	-3.797	58
0.438	-3.792	59
0.723	-3.757	60
0.638	-3.742	61
0.543	-3.717	62
0.702	-3.700	63
0.583	-3.691	64
0.646	-3.586	65
0.457	-3.580	66
0.787	-3.524	67
0.765	-3.510	68
0.686	-3.491	69
0.811	-3.429	70
0.778	-3.423	71
0.729	-3.389	72
0.489	-3.389	73
0.604	-3.263	74
0.574	-3.213	75
0.688	-3.178	76
0.587	-3.150	77
0.681	-3.103	78
0.673	-2.930	79
0.660	-2.835	80
0.729	-2.782	81
0.592	-2.771	82
0.750	-2.760	83
0.532	-2.733	84
0.660	-2.732	85
0.458	-2.722	86
0.681	-2.620	87

0.712	-2.499	88
0.681	-2.485	89
0.478	-2.438	90
0.712	-2.426	91
0.574	-2.371	92
0.500	-1.821	93
0.735	-1.220	94
0.776	-0.556	95

Table S7. Our hit compounds ranked by Docking score (XP GScore) with Tanimoto score based on the similarity with PDB 3AVG

Canvas Tanimoto Similarity	XP GScore	Fragment number
0.661	-5.546	96
0.464	-4.834	97
0.589	-4.419	98
0.554	-4.269	99
0.875	-4.228	100
0.554	-4.193	101
0.643	-4.121	102
0.679	-4.012	103
0.571	-3.864	104
0.554	-3.848	105
0.518	-3.784	106
0.446	-3.638	107
0.607	-3.526	108
0.696	-3.524	109
0.571	-3.511	110
0.804	-3.510	111
0.732	-3.443	112
0.393	-3.250	113
0.571	-3.221	114
0.500	-3.217	115
0.464	-3.181	116
0.804	-3.161	117
0.625	-3.142	118
0.446	-3.131	119
0.518	-3.089	120
0.786	-3.014	121
0.464	-2.966	122
0.464	-2.931	123
0.661	-2.930	124
0.429	-2.929	125
0.482	-2.878	126
0.589	-2.835	127
0.679	-2.782	128
0.571	-2.713	129
0.786	-2.676	130
0.446	-2.667	131
0.536	-2.645	132
0.732	-2.624	133
0.786	-2.623	134
0.643	-2.455	135
0.696	-2.442	136
0.750	-2.405	137

0.589	-2.382	138
0.607	-2.367	139
0.643	-2.302	140
0.750	-2.222	141
0.393	-1.964	142
0.821	-1.820	143
0.750	-1.302	144
0.661	-4.761	145
0.589	-4.419	146
0.554	-4.390	147
0.446	-4.377	148
0.679	-4.110	149
0.554	-4.058	150
0.679	-4.012	151
0.930	-3.801	152
0.589	-3.797	153
0.429	-3.792	154
0.643	-3.757	155
0.571	-3.742	156
0.464	-3.717	157
0.625	-3.700	158
0.554	-3.691	159
0.607	-3.586	160
0.393	-3.580	161
0.696	-3.524	162
0.804	-3.510	163
0.732	-3.491	164
0.911	-3.429	165
0.911	-3.423	166
0.679	-3.389	167
0.446	-3.389	168
0.571	-3.263	169
0.518	-3.213	170
0.643	-3.178	171
0.500	-3.150	172
0.607	-3.103	173
0.661	-2.930	174
0.589	-2.835	175
0.679	-2.782	176
0.589	-2.771	177
0.821	-2.760	178
0.482	-2.733	179
0.589	-2.732	180
0.446	-2.722	181
0.607	-2.620	182
0.786	-2.499	183

0.607	-2.485	184
0.411	-2.438	185
0.786	-2.426	186
0.518	-2.371	187
0.429	-1.821	188
0.714	-1.220	189
0.750	-0.556	190

Table S8. Our hit compounds ranked by Docking score (XP GScore) with Tanimoto score based on the similarity with PDB 4CFD

Canvas Tanimoto Similarity	XP GScore	Fragment number
0.895	-5.546	191
0.694	-4.834	192
0.838	-4.419	193
0.833	-4.269	194
0.615	-4.228	195
0.833	-4.193	196
0.868	-4.121	197
0.872	-4.012	198
0.914	-3.864	199
0.833	-3.848	200
0.778	-3.784	201
0.667	-3.638	202
0.865	-3.526	203
0.721	-3.524	204
0.811	-3.511	205
0.702	-3.510	206
0.854	-3.443	207
0.629	-3.250	208
0.861	-3.221	209
0.750	-3.217	210
0.694	-3.181	211
0.778	-3.161	212
0.892	-3.142	213
0.667	-3.131	214
0.829	-3.089	215
0.717	-3.014	216
0.694	-2.966	217
0.694	-2.931	218
0.946	-2.930	219
0.686	-2.929	220
0.722	-2.878	221
0.943	-2.835	222
0.780	-2.782	223
0.811	-2.713	224
0.795	-2.676	225
0.667	-2.667	226
0.806	-2.645	227
0.767	-2.624	228
0.717	-2.623	229
0.868	-2.455	230
0.850	-2.442	231
0.750	-2.405	232

0.838	-2.382	233
0.725	-2.367	234
0.690	-2.302	235
0.750	-2.222	236
0.629	-1.964	237
0.723	-1.820	238
0.711	-1.302	239
0.895	-4.761	240
0.838	-4.419	241
0.833	-4.390	242
0.667	-4.377	243
0.921	-4.110	244
0.833	-4.058	245
0.872	-4.012	246
0.618	-3.801	247
0.889	-3.797	248
0.639	-3.792	249
0.868	-3.757	250
0.914	-3.742	251
0.694	-3.717	252
0.892	-3.700	253
0.833	-3.691	254
0.683	-3.586	255
0.629	-3.580	256
0.721	-3.524	257
0.702	-3.510	258
0.854	-3.491	259
0.623	-3.429	260
0.654	-3.423	261
0.659	-3.389	262
0.667	-3.389	263
0.861	-3.263	264
0.829	-3.213	265
0.775	-3.178	266
0.750	-3.150	267
0.971	-3.103	268
0.946	-2.930	269
0.943	-2.835	270
0.780	-2.782	271
0.838	-2.771	272
0.723	-2.760	273
0.722	-2.733	274
0.789	-2.732	275
0.667	-2.722	276
0.769	-2.620	277
0.717	-2.499	278

0.725	-2.485	279
0.657	-2.438	280
0.717	-2.426	281
0.778	-2.371	282
0.686	-1.821	283
0.744	-1.220	284
0.711	-0.556	285
